# COX4-1 promotes mitochondrial supercomplex assembly and limits reactive oxide species production in radioresistant GBM

**DOI:** 10.15698/cst2022.04.266

**Published:** 2022-03-07

**Authors:** Claudia R. Oliva, Md Yousuf Ali, Susanne Flor, Corinne E. Griguer

**Affiliations:** 1 Free Radical & Radiation Biology Program, Department of Radiation Oncology, The University of Iowa, Iowa City, IA 52242.; 2 Interdisciplinary Graduate Program in Human Toxicology, The University of Iowa, Iowa City, IA 52242.

**Keywords:** supercomplexes, cytochrome c oxidase, radioresistance, GBM, superoxide, COX4, mitochondria

## Abstract

Glioblastoma (GBM) is a fatal disease with recurrences often associated with radioresistance. Although often effective at treating newly diagnosed GBM, increasing evidence suggests that radiotherapy-induced alterations in tumor metabolism promote GBM recurrence and aggressiveness. Using isogenic radiosensitive and radioresistant GBM cell lines and patient-derived xenolines, we found that acquired radioresistance is associated with a shift from a glycolytic metabolism to a more oxidative metabolism marked by a substantial increase in the activity of the mitochondrial respiratory chain complex cytochrome c oxidase (CcO). This elevated CcO activity was associated with a switch in the isoform expression of the CcO regulatory subunit COX4, from COX4-2 to COX4-1, assembly of CcO-containing mitochondrial supercomplexes (SCs), and reduced superoxide (O_2_^•-^) production. Overexpression of COX4-1 in the radiosensitive cells was sufficient to promote the switch from glycolytic to oxidative metabolism and the incorporation of CcO into SCs, with a concomitant reduction in O_2_^•-^ production. Conversely, silencing of COX4-1 expression in normally radioresistant cells reduced CcO activity, promoted the disassembly of mitochondrial SCs, and increased O_2_^•-^ production. Additionally, gain or loss of COX4-1 expression was sufficient to induce the radioresistant or radiosensitive phenotype, respectively. Our results demonstrate that COX4-1 promotes SC assembly in GBM cells, and SC assembly may in turn regulate the production of reactive oxygen species and thus the acquisition of radioresistance in GBM.

## INTRODUCTION

Glioblastoma (GBM) is the most common and aggressive primary brain tumor in adults [1]. Radiotherapy is a critical treatment modality for newly diagnosed GBM [2] but is also the primary cause of tumor recurrence and death in these patients [3]. Despite recent technological advances, the development of acquired resistance to therapeutic radiation continues to present a major clinical hurdle in the treatment of GBM. Currently it is not possible to predict in whom or when resistance to radiotherapy will develop [4, 5], and the molecular mechanisms underlying this resistance have not been well defined.

It has long been recognized that perturbed cellular metabolism is a hallmark of many cancer cells [6–11]. However, the significance of metabolic changes in resistance to therapy are only now being realized [12]. Interestingly, various therapy-resistant cancer cells show a high dependence on mitochondrial respiratory function and the consequent metabolic phenotype [10, 11, 13–15]. Researchers have long believed that cancer cells rely exclusively on glycolytic metabolism. However, accumulating evidence has begun to show that oxidative phosphorylation (OXPHOS) is critically involved in tumor progression and resistance to therapy [16, 17]. Notably, a small number of studies have demonstrated a shift in cancer cell metabolism toward OXPHOS in chemo- and radioresistant cancer cells [10, 11, 18, 19].

Despite these recent findings, whether and how changes in mitochondrial function and metabolic phenotype affect cancer cell sensitivity to radiotherapy remain largely unclear, especially regarding GBM. Evidence accumulated in the last ten years has demonstrated that, rather than functioning solely as individual units, mitochondrial respiratory chain complexes, especially complexes I, III, and IV (cytochrome c oxidase; CcO), often interact to form supercomplexes (SCs), also referred to as respirasomes [20–25]. Although the functional significance of mitochondrial SCs is just beginning to emerge, it appears that these structures enhance electron transport (i.e., OXPHOS efficiency), which in turn may reduce the rate of reactive oxygen species (ROS) generation [26, 27]. Complexes I and III are the major redox centers in which O_2_ is reduced to superoxide (O_2_^•-^) within the mitochondrial respiratory chain, and complex III appears to be the major site of radiation-induced ROS production [28, 29]. Therefore, it was proposed that SC assembly of these complexes minimizes ROS production [30]. Confirming this idea in an *ex vivo* biological system, Lopez-Fabuel *et al.* found that the abundance of free complex I relative to the abundance of complex I in SCs is higher in astrocytes than in neurons, and this high abundance of free complex I in astrocytes correlates with higher ROS production [31]. As tumor cell production of ROS is necessary for radiation-induced cell death, SC assembly may be a factor in determining tumor cell radiosensitivity. However, the current understanding of how SCs are assembled and regulated, and what their physiological function in health and disease is, remains limited [23, 32–34].

Recently, several reports have suggested that specific subunits of CcO (complex IV) regulate SC formation [21, 27, 35, 36]. CcO is the terminal enzyme of the mitochondrial respiratory chain and, as such, CcO activity controls ATP production and OXPHOS efficiency in mammalian cells. The three mitochondria-encoded subunits of CcO are necessary for the catalytic function, while the eleven nuclear-encoded subunits regulate the enzymatic activity of CcO [37]. COX4, the largest regulatory subunit of CcO, has been shown to inhibit the enzymatic activity of CcO when ATP concentrations are high [38]. The two isoforms of COX4, namely COX4-1 and COX4-2, share high homology in the C-terminal region but differ significantly in the N-terminal region, which comprises the matrix domain. Additionally, COX4-2 lacks the regulatory S58 residue involved in the regulation of ATP binding [39] but contains three cysteine residues that have been suggested to function as redox sensors [40]. We previously demonstrated that expression of the COX4-1 isoform is associated with elevated CcO activity and chemoresistance in GBM cells [11, 15]. However, the involvement of COX4-1 expression in SC assembly, metabolic phenotype, and the mechanism of acquired radioresistance in GBM has not been examined.

Here, we investigated the metabolic phenotype of radiosensitive parental cell lines and derived cells with acquired radioresistance. Furthermore, we examined the relationship between SC assembly and metabolic phenotype as well as the molecular mechanisms that regulate SC assembly and the acquisition of radioresistance in GBM cells.

## RESULTS

### Acquired radioresistance in GBM cells is associated with a switch from glycolytic to oxidative metabolism

**Figure 1:**
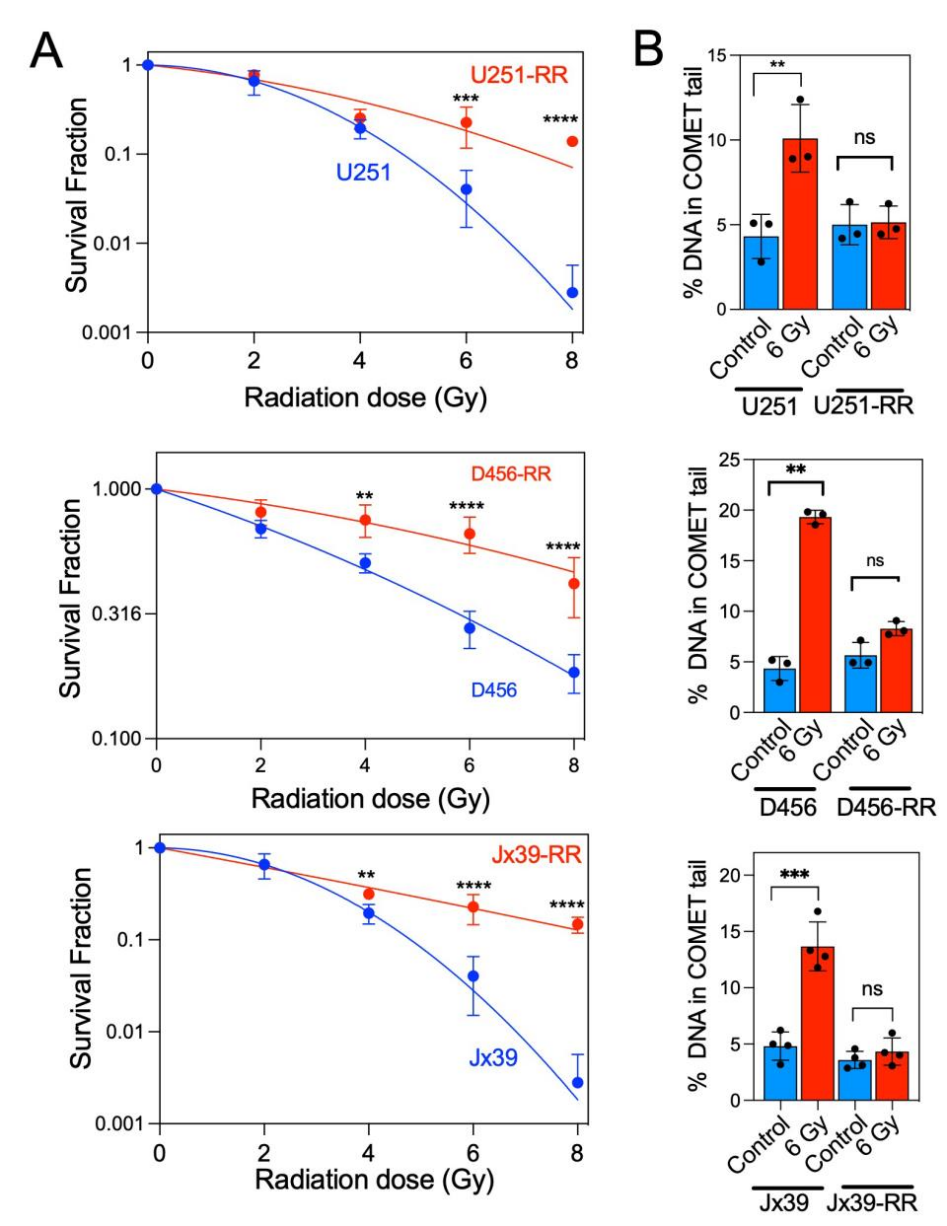
FIGURE 1: Fractionated radiation promotes acquired radioresistance in GBM cell lines. **(A)** Clonogenic survival curves for radiosensitive (U251, D456, and Jx39) and isogenic radioresistant (U251-RR, D456-RR and Jx39-RR) cell lines. Cells were irradiated with 2, 4, 6, or 8 Gy and immediately plated. Clonogenic survival was estimated on day 14 after irradiation. **(B)** Quantification of post-irradiation DNA damage in the radiosensitive and radioresistant GBM cells, assessed by single-cell gel electrophoresis assay under neutral conditions (neutral comet assay). Data are presented as the mean ± SEM (n=3). p < 0.01 (**), p < 0.001 (***), and p < 0.0001 (****), calculated by Student t-test. ns, not significant.

To investigate the role of mitochondrial alterations in the radioresistance of GBM cells, we generated isogenic GBM cell models of radioresistance by exposing U251 cells and patient-derived xenograft lines (D456 and Jx39) to chronic irradiation with clinically relevant fractionated doses (5 Gy) of X-ray radiation, delivered once weekly to a cumulative dose of 25 Gy. After the radioresistant lines were established, the three pairs of radioresistant and parental cell lines were treated with radiation doses between 2 and 8 Gy. The survival rate after irradiation was significantly higher in the isogenic radioresistant sublines (U251-RR, D456-RR, and Jx39-RR) than in the corresponding radiosensitive cell lines (**Fig. 1A**). The mean inactivation dose (MID, dose causing 50% cell death) was calculated for each cell line by fitting the data to the linear-quadratic model [41]. The MID ratio (ratio of radioresistant to radiosensitive MID) was 1.58, 1.97, and 2.31 for U251, D456, and Jx39 cells, respectively (p < 0.001 in each case). Consistently, comet assays conducted 24 h after cells were irradiated with 6 Gy showed only a low level of DNA damage in the isogenic radioresistant lines but heavy damage in the radiosensitive cell lines (**Fig. 1B**). These paired cell lines of the same origin but with distinctly different radiosensitivity thus provide unique models with which to investigate metabolic changes associated with acquired radioresistance.

**Figure 2:**
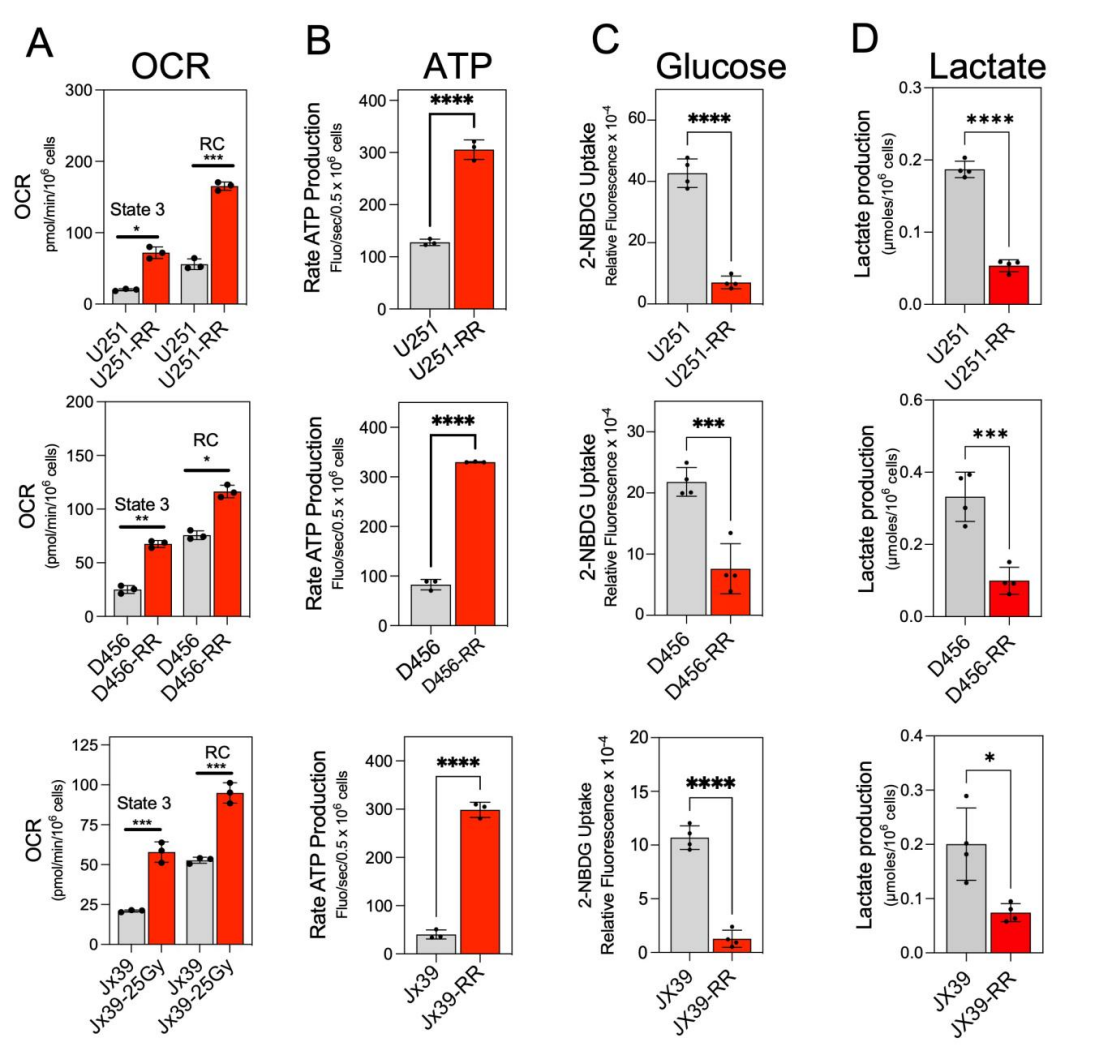
FIGURE 2: Acquired radioresistance in GBM cells is associated with a switch to an OXPHOS phenotype. **(A)** Quantification of the OCR in radiosensitive (U251, D456, and Jx39) and isogenic radioresistant (U251-RR, D456-RR, and Jx39-RR) cell lines after the addition of pyruvate/malate and ADP (state 3) and after the subsequent addition of FCCP (reserve capacity). Data are presented as the mean ± SEM (n=3). **(B)** Quantification of ATP production rate in radiosensitive and radioresistant cell lines, assessed by the reduction of NADP^+^ to NADPH. Data are presented as the mean ± SEM (n=3). **(C)** Quantification of cellular glucose uptake in the radiosensitive and radioresistant cell lines estimated with the non-phosphorylatable fluorescent glucose analogue 2-NBDG. Indicated cells were incubated with 100 μM 2-NBDG for 20 min, and 2-NBDG fluorescence was measured in a microplate fluorometer. Data are presented as the mean ± SEM (n= 4). **(D)** Quantification of extracellular lactate concentrations in cultures of the radiosensitive and radioresistant cell lines. Data are presented as the mean ± SEM (n= 4). OCR, oxygen consumption rate; Res capacity, reserve capacity; 2-NBDG, 2-(N-(7-nitrobenz-2-oxa-1,3-diazol-4-yl)amino)-2-deoxyglucose. p < 0.05 (*), p < 0.01 (**), p < 0.001 (***), and p < 0.0001 (****), calculated by Student t-test.

We next assessed the metabolic phenotype of radioresistant and radiosensitive GBM cells, determining the mitochondrial oxygen consumption rate (OCR) and ATP production rate to reflect mitochondrial OXPHOS activity and the extent of cellular glucose uptake and lactate production to reflect glycolytic activity [21, 42]. OCR was determined in isolated mitochondria after activation of complex I driven by addition of pyruvate and malate, followed by ADP (state 3 respiration). As shown in **Figure 2A**, state 3 respiration was significantly higher in the mitochondria from the isogenic radioresistant cell lines than in the mitochondria from the corresponding radiosensitive lines. To determine the maximal mitochondrial uncoupled respiration rate (reserve capacity), carbonyl cyanide p-trifluoromethoxy-phenylhydrazone (FCCP) was sequentially added at increasing concentrations, and maximal uncoupled respiration was obtained at 1.0 µM FCCP. The reserve capacity was also significantly higher in the mitochondria from the isogenic radioresistant cell lines than in the mitochondria of the corresponding radiosensitive cell lines (**Fig. 2A**).

In the OXPHOS phenotype, higher OCR is linked to higher cellular energy production [43]. Therefore, to further confirm that the induction of radioresistance in GBM cells is associated with a switch to the OXPHOS phenotype, we examined the rate of ATP production in each cell line.

The rate of ATP production was measured by coupling ATP hydrolysis to the reduction of NADP^+^ to NADPH [44]. As shown in **Figure 2B**, the rate of ATP production was 3- to 4-fold higher in the isogenic radioresistant lines than in the corresponding radiosensitive cell lines. In contrast, cellular glucose consumption and lactate production were lower in the radioresistant cells than in the corresponding radiosensitive cell lines (**Fig. 2C-D**). These results indicate that acquired radioresistance in GBM cells is associated with increased OXPHOS and decreased glycolysis.

### CcO is incorporated into mitochondrial SCs in radioresistant GBM cells.

**Figure 3:**
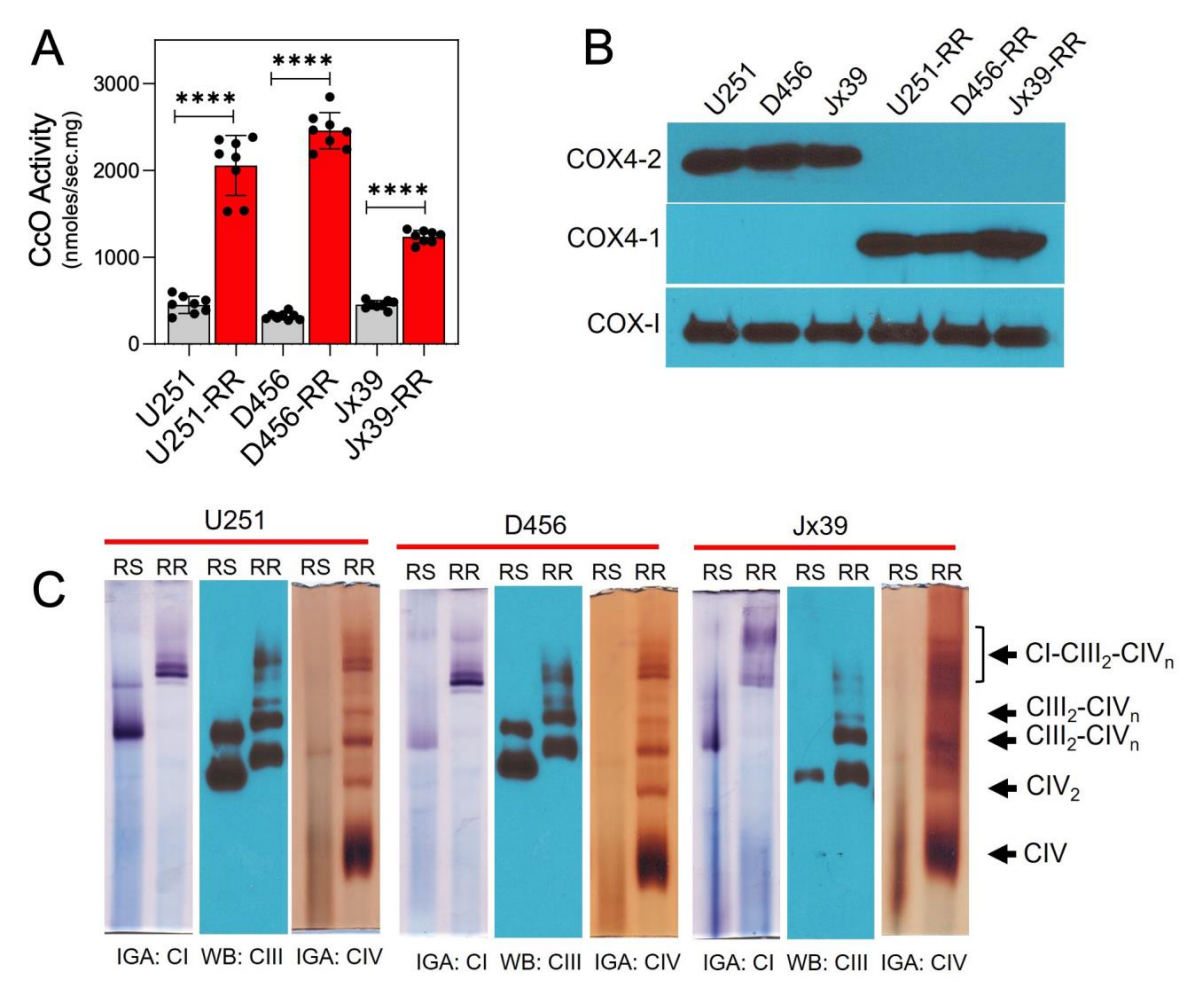
FIGURE 3: CcO is incorporated into mitochondrial SCs in GBM cells with acquired radioresistance. **(A)** CcO activity in radiosensitive (U251, D456, and Jx39) and isogenic radioresistant (U251-RR, D456-RR, and Jx39-RR) cell lines. Data are presented as the mean ± SEM (n=4). **(B)** Representative Western blot showing the expression of the COX4 isoforms COX4-1 and COX4-2 in the radiosensitive and radioresistant cells. COX-I was probed as a loading control. **(C)** Digitonin-solubilized mitochondria from radiosensitive and radioresistant cell lines were subjected to BN-PAGE followed by complex I and IV IGA assays and Western blot for complex III. Representative image from 3 separate experiments. p < 0.0001 (****), calculated by Student t-test.

Because CcO has been shown to have an important role in the regulation of electron transport and OXPHOS [11, 30, 44, 45], we compared CcO activity in mitochondria isolated from the parental radiosensitive and isogenic radioresistant GBM cell lines. CcO activity was significantly higher in the radioresistant cells (2056.0 ± 122.3, 2456.0 ± 17.2, and 1236 ± 25.1 nmol/sec/mg for U251-RR, D456-RR, and Jx39-RR cells, respectively) than in the radiosensitive cells (450.5 ± 35.5, 319.4 ± 14.8, and 452.5 ± 17.2 nmol/sec/mg for U251, D456, and Jx39 cells, respectively; *p* <0.0001 for each cell line pair; **Fig. 3A**). We [10, 11, 15] and others [46–49] have demonstrated that the nuclear-encoded COX4 isoforms are key regulatory subunits of mammalian CcO and could have a role in GBM resistance to therapy. Western blot analysis of COX4 isoform expression in our cell lines indicated that COX4-1 is expressed almost exclusively in the isogenic radioresistant cells, whereas COX4-2 is expressed almost exclusively in the corresponding radiosensitive cells (**Fig. 3B**).

CcO-specific subunit isoforms have been shown to influence SC assembly as well [21, 27, 35, 36]. Therefore, we assessed whether the structural organization of the mitochondrial respiratory chain differs between the radiosensitive and isogenic radioresistant GBM cell lines. To test this possibility, digitonin-solubilized mitochondria from radiosensitive and radioresistant cell lines were subjected to blue native PAGE (BN-PAGE), which facilitates the separation of respiratory chain complex monomers and SCs. In mitochondria from the radioresistant cells, CcO (complex IV) was detected as dimers or in SCs (**Fig. 3C**). Specifically, CcO was identified at different copy numbers (n) in complex III-IV SCs (III_2_IV_n_) and in larger complex I-III-IV SCs (I_1_III_2_IV_n_). In mitochondria from the isogenic radiosensitive cells, CcO was detected in monomeric form and in SCs with complex III, but respirasomes were not detected. In agreement with the *in vitro* determination of CcO activity (**Fig. 3A**), CcO activity detected via in-gel activity (IGA) assay was significantly lower in samples from the radiosensitive cells than in samples from the radioresistant cells. An association between complex I and complex III was also apparent in the radiosensitive cell lines (I_1_-III_2_; **Fig. 3C**).

### Radiosensitive cells produce more mitochondrial ROS than radioresistant cells do

**Figure 4:**
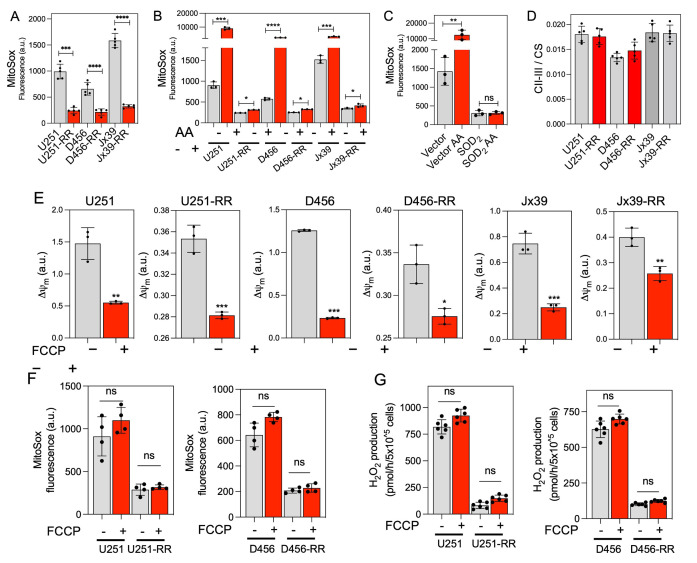
FIGURE 4: ROS production is lower in GBM cells with acquired radioresistance. **(A)** Quantification of mitochondrial O_2_^•-^ production in radiosensitive (U251, D456, and Jx39) and isogenic radioresistant (U251-RR, D456-RR, and Jx39-RR) cell lines, determined by MitoSOX assays. Data are presented as the mean ± SEM (n=4). **(B)** Quantification of mitochondrial O_2_^•-^ production in the radiosensitive and radioresistant cells treated without or with AA (10 μM, 15 min) to inhibit complex III. Data are presented as the mean ± SEM (n=3). **(C)** Quantification of mitochondrial O_2_^•-^ production in vector control- and SOD_2_-transfected U251 cells treated without or with AA (10 μM, 15 min). Data are presented as the mean ± SEM (n=2). **(D)** Quantification of complex II/III activity in radiosensitive and radioresistant cells. Data are presented as the mean ± SEM (n=3). **(E)** Quantification of mitochondrial membrane potential (Δψm) and O_2_^•-^ production **(F)** in radiosensitive and radioresistant cells treated without or with the uncoupler FCCP (10 μM, 15 min). Data are presented as the mean ± SEM (n=3). **(G)** Quantification of mitochondrial H_2_O_2_ production, determined by Amplex Red oxidation assays, in radiosensitive and radioresistant cells treated without or with the uncoupler FCCP (10 μM, 15 min). Data are presented as the mean ± SEM (n=2). p < 0.05 (*), p < 0.01 (**), p < 0.001 (***) and p < 0.0001 (****), respectively, calculated by Student t-test. a.u., arbitrary units; ns, not significant; CS, citrate synthase; Δψ_m,_ mitochondrial membrane potential; and FCCP, carbonyl cyanide p-trifluoromethoxyphenylhydrazone.

The organization of respiratory complexes into SCs has been reported to affect mitochondrial function and ROS production [26, 31, 44]. Because there is unequivocal evidence that ROS influence the genotoxic stress caused by ionizing radiation [50, 51], we next used the mitochondria-targeted probe MitoSOX to determine O_2_^•-^ production in intact radiosensitive and isogenic radioresistant GBM cells. O_2_^•-^ production was detected in all cell lines but was 3- to 5-fold higher in the radiosensitive cells than in the corresponding radioresistant cells under control conditions (**Fig. 4A**). Mitochondrial complex III has been identified as the main producer of O_2_^•-^ and derived ROS within the respiratory chain [52, 53] and is considered the major site of ROS production after irradiation [28, 29]. Inhibition of complex III with antimycin A (AA) enhances O_2_^•-^ production [54]. Notably, mitochondrial O_2_^•-^ production stimulated by the C-III inhibitor AA was significantly attenuated in radioresistant cells compared with radiosensitive cells (**Fig. 4B**). In the radiosensitive U251 cells overexpressing the antioxidant superoxide dismutase 2 (SOD_2_), only a very low MitoSOX signal was detected before or after treatment with AA, confirming the specificity of the MitoSOX probe for O_2_^•-^ (**Fig. 4C**). The activity of complexes II-III was similar in the radiosensitive and radioresistant lines under basal conditions, indicating that the differences in O_2_^•-^ production were not due to a depletion of complex III in the radioresistant cells (**Fig. 4D**). No significant differences in MitoSOX fluorescence were observed after mitochondrial membrane potential disruption with FCCP (**Fig. 4E** and **F**), suggesting that the differences in MitoSOX fluorescence were not due to differences in mitochondrial membrane potential affecting probe uptake. We also determined cellular levels of H_2_O_2_ production by monitoring the oxidation of Amplex Red. H_2_O_2_ production was about four orders of magnitude faster in the radiosensitive cells than in the corresponding radioresistant cells, and no significant differences in H_2_O_2_ production were detected between the cell lines after mitochondrial membrane potential disruption with FCCP (**Fig. 4G**).

### COX4-1 promotes SC assembly and modulates ROS production

**Figure 5:**
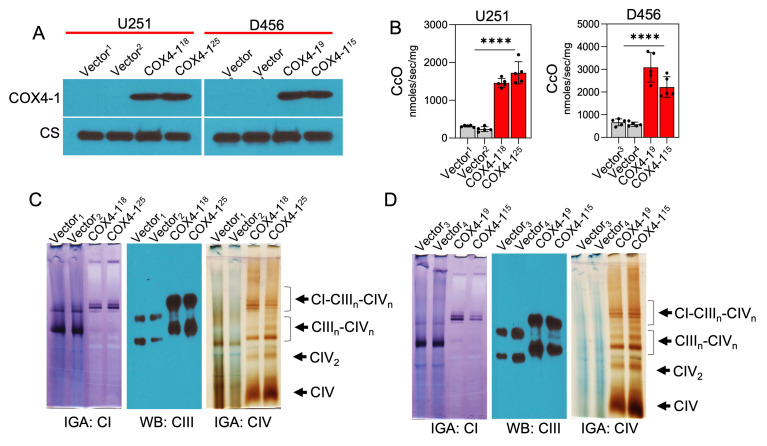
FIGURE 5: COX4-1 overexpression promotes SC assembly in radiosensitive GBM cells. **(A)** Representative Western blot showing COX4-1 expression in U251 and D456 cells stably transfected with vector only or vector expressing COX4-1. Citrate synthase (CS) served as the loading control. **(B)** Quantification of CcO activity in vector control- and COX4-1-transfected U251 and D456 cells. Data are presented as the mean ± SEM (n=4). **(C)** Digitonin-solubilized mitochondria from the indicated cell lines were subjected to BN-PAGE followed by in-gel complex I and IV activity assays and complex III Western blot analysis. Representative images from 3 separate experiments. p < 0.0001 (****), calculated by Student t-test.

Considering that COX4-1 expression was associated with the presence of SCs, we further tested the effects of COX4-1 overexpression on CcO activity and SC assembly. For this purpose, we used stable U251 and D456 cell lines ectopically expressing FLAG-tagged COX4-1 (COX4-1-U251 and COX4-1-D456, respectively) or the FLAG expression vector (vector-U251 and vector-D456, respectively; **Fig. 5A**). CcO activity was significantly higher in COX4-1-overexpressing glioma cells (1728.0 ± 131.7 nmol/sec/mg and 1460.0 ± 51.3 nmol/sec/mg in COX4-1-U251 clones versus 316.5 ± 11.0 nmol/sec/mg and 236.9 ± 28.8 nmol/sec/mg in vector-U251 clones; 3087.0 ± 290.7 nmol/sec/mg and 2220.0 ± 204.5 nmol/sec/mg in COX4-1-D456 clones versus 663.6 ± 68.2 nmol/sec/mg and 581.0 ± 42.78 nmol/sec/mg in vector-D456 clones; *p* <0.0001; **Fig. 5B**).

**Figure 6:**
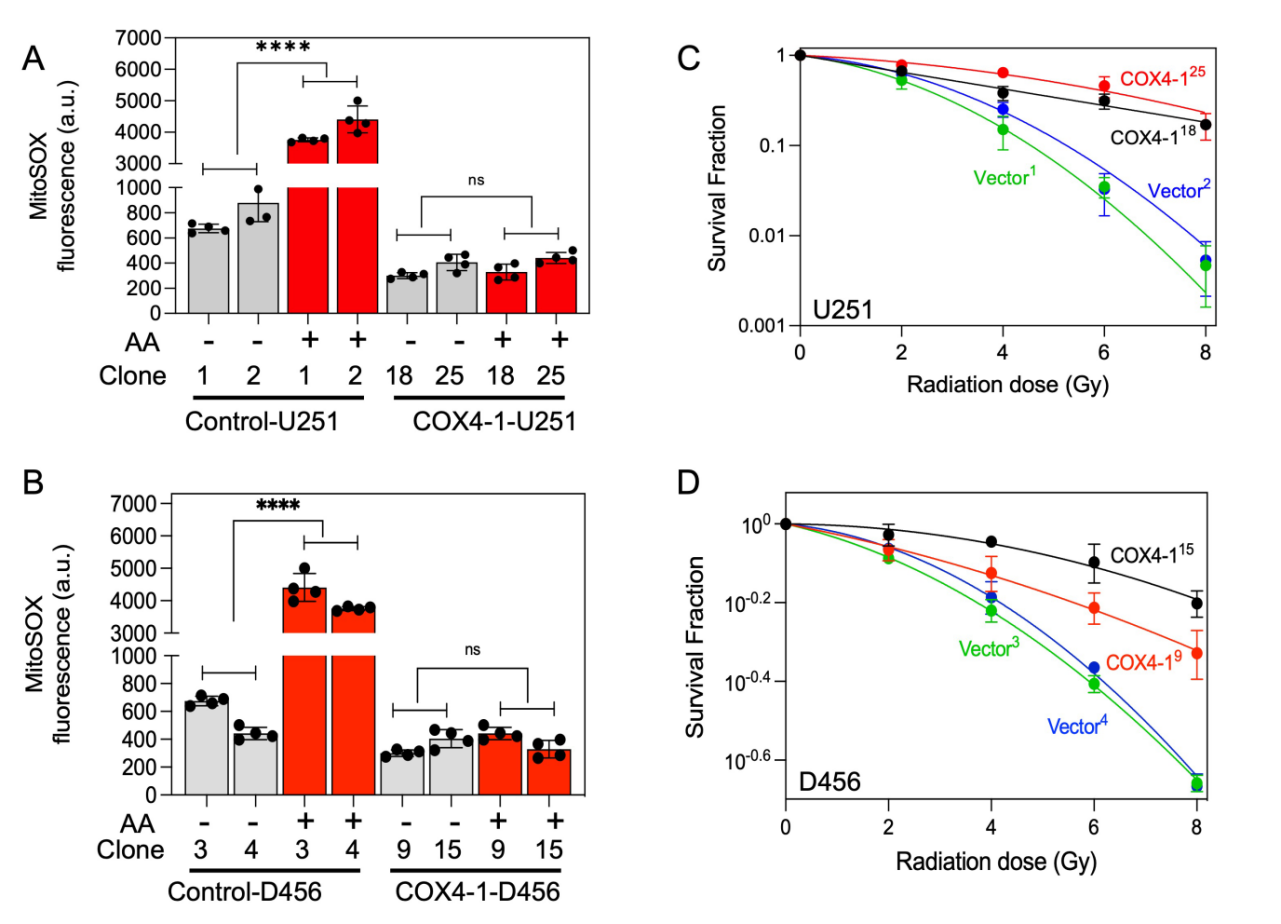
FIGURE 6: O_2_^•-^ production is reduced in COX4-1 overexpressing GBM cells. Quantification of mitochondrial O_2_^•-^ production in vector-transfected controls and COX4-1-overexpressing U251 cells **(A)** and D456 **(B)** cells treated with or without AA (10 μM for 15 min), assessed by MitoSOX assay. Data are presented as the mean ± SEM (n= 3). Clonogenic survival curves for vector-transfected controls and COX4-1-overexpressing U251 **(C)** and D456 **(D)** cells. Cells were irradiated with 2, 4, 6, or 8 Gy and immediately plated. Clonogenic survival was estimated on day 14 after irradiation. p < 0.01 (**) and p < 0.001 (***), calculated using one-way ANOVA followed by Tukey's multiple comparison test. p < 0.0001 (****), calculated by Student t-test. a.u., arbitrary units; AA, antimycin A; ns, not significant.

To investigate the effect of COX4-1 overexpression on SC assembly, mitochondria prepared from COX4-1-overexpressing and vector-overexpressing U251 and D456 cells were analyzed by BN-PAGE with subsequent IGA assays or immunoblotting. The formation of CcO-containing SCs was higher in COX4-1-overexpressing U251 and D456 clones than in the respective vector-transfected clones. The results showed that overexpression of COX4-1 decreased the signal of monomeric complex I and complex III but increased the signal at the position of SCs (III_n_-IV_n_ and I-III_n_-IV_n_; **Fig. 5C** and **D**). Because SCs are hypothesized to enhance electron transport activity without a compensatory increase in ROS, we analyzed the mitochondrial ROS levels in COX4-1-overexpressing cells. Indeed, O_2_^•-^ levels were lower in COX4-1-U251 (**Fig. 6A**) and COX4-1-D456 (**Fig. 6B**) clones than in vector-control clones and, as in radioresistant cells, O_2_^•-^ levels did not increase after AA treatment in COX4-1-overexpressing clones (**Fig.6A** and **B**).

We next investigated whether the overexpression of COX4-1 is sufficient to enhance radioresistance in GBM cells. After irradiation with up to 8 Gy, clonogenic survival was significantly higher in U251-COX4-1 (**Fig. 6C**) and D456-COX4-1(**Fig. 6D**) clones than in the respective vector-transfected U251 and D456 clones.

### Depletion of COX4-1 disrupts SC assembly and promotes ROS production

**Figure 7:**
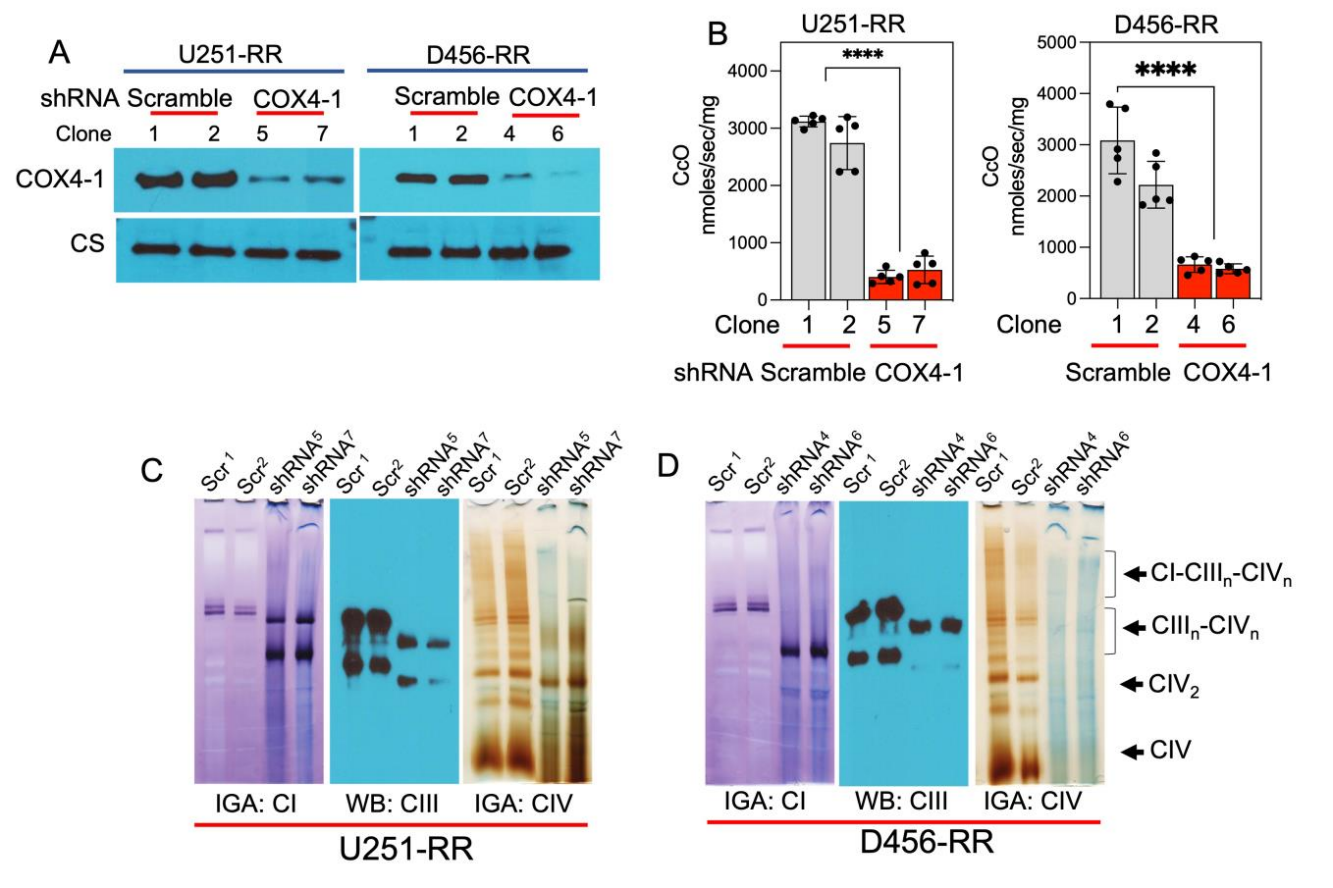
FIGURE 7: Silencing COX4-1 reduces SC assembly in in GBM cells with acquired radioresistance. **(A)** Representative Western blot showing COX4-1 expression in U251-RR and D456-RR cells transfected with shRNA targeting COX4-1 or scramble shRNA. Citrate synthase (CS) served as the loading control. **(B)** Quantification of CcO activity in in U251-RR and D456-RR cells transfected with shRNA targeting COX4-1 or scramble shRNA. Data are presented as the mean ± SEM (n=3) **(C)** Digitonin-solubilized mitochondria from the indicated cell lines were subjected to BN-PAGE followed by complex I and IV IGA assays and complex III Western blot analysis. Representative images from 4 independent preparations. p < 0.0001 (****), calculated by Student t-test.

We next examined the consequences of COX4-1 depletion on CcO activity, SC assembly, ROS production, and resistance to radiation. Western blot analysis showed that COX4-1 protein expression was significantly attenuated in U251-RR and D456-RR cells transfected with shRNA targeting *COX4i1* (COX4-1 sgRNA; **Fig. 7A**). Furthermore, CcO activity was significantly reduced in these COX4-1-depleted U251-RR and D456-RR cells (404.0 ± 51.7 nmol/sec/mg and 527.8 ± 107.7 nmol/sec/mg for COX4-1-shRNA-U251-RR clones versus 3118.0 ± 41.9 nmol/sec/mg and 2742.0 ± 206.5 nmol/sec/mg for scramble shRNA-U251-RR clones; 3087.0 ± 290.7 nmol/sec/mg and 2220.0 ± 204.5 nmol/sec/mg for scramble-shRNA-D456-RR clones versus 663.6 ± 68.2 nmol/sec/mg and 581.0 ± 42.8 nmol/sec/mg for COX4-1 shRNA-D456-RR clones; p < 0.0001; **Fig. 7B**).

**Figure 8:**
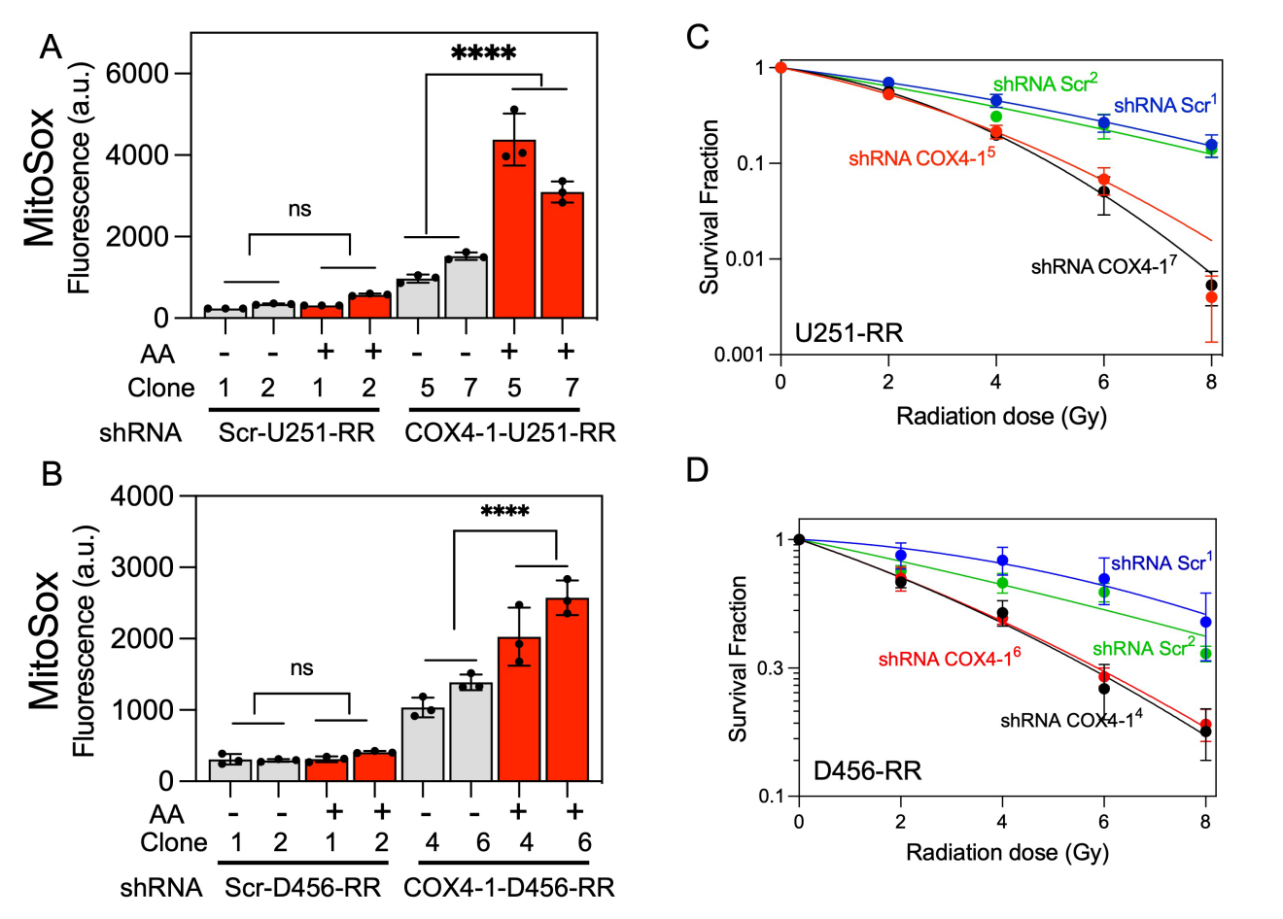
FIGURE 8: Silencing COX4-1 increases O_2_^•-^ production in GBM cells with acquired radioresistance. **(A)** Quantification of mitochondrial O_2_^•-^ production in U251-RR (top) and D456-RR (bottom) cells transfected with shRNA targeting COX4-1 or scramble shRNA and treated with or without AA (10 μM for 15 min), assessed by MitoSOX assay. Data are presented as the mean ± SEM (n=3). **(B)** Clonogenic survival curves for U251-RR (top) and D456-RR (bottom) cells transfected with shRNA targeting COX4-1 or scramble shRNA. Cells were irradiated (0–8 Gy) and immediately plated. Clonogenic survival was estimated on day 14 after irradiation. ***p<0.001, calculated using one-way ANOVA followed by Tukey's multiple comparison test. p < 0.0001 (****), calculated by Student t-test. a.u., arbitrary units; AA, antimycin A; ns, not significant.

To analyze mitochondrial SC assembly in COX4-1-depleted cells, we performed BN-PAGE followed by IGA assays or immunoblotting in digitonin-solubilized mitochondria from COX4-1-shRNA-U251-RR cells and scramble shRNA-U251-RR cells. Depletion of COX4-1 appeared to shift the distribution of complexes I, III, and IV from SCs to monomers (**Fig. 7C**), and this trend was maintained when COX4-1 protein expression was silenced in D456-RR cells (**Fig. 7D**). In addition, O_2_^•-^ levels were higher in the COX4-1-shRNA-U251-RR cells (3- to 5-fold) and COX4-1-shRNA-D456-RR cells (3- to 4-fold) than in scramble shRNA-transfected counterparts. Notably, treatment with AA further increased O_2_^•-^ levels in COX4-1-shRNA-U251-RR cells (5- to 7-fold) and COX4-1-shRNA-D456-RR cells (6- to 10-fold) but not in scramble shRNA-transfected counterparts (**Fig. 8A** and **B**). Finally, clonogenic survival was significantly lower in COX4-1-depleted U251-RR and D456-RR clones than in scramble shRNA-transfected clones after irradiation with up to 8 Gy (**Fig. 8C** and **D**).

### CcO is incorporated into mitochondrial SCs in human GBM tumors

**Figure 9:**
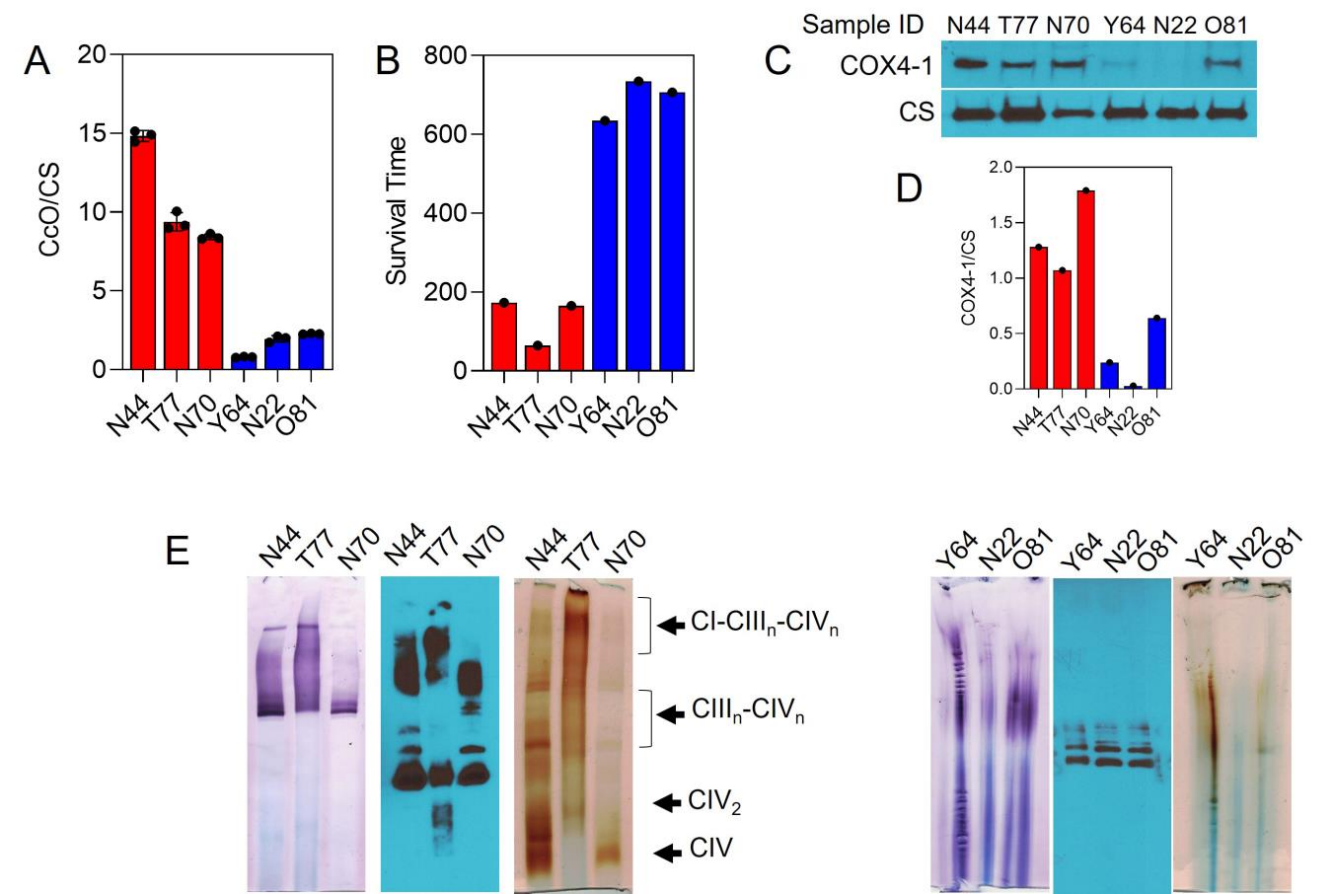
FIGURE 9: CcO is incorporated into mitochondrial SCs in patient biopsy samples. **(A)** CcO activity in human GBM tumors. Data are presented as the mean ± SEM (n=3). **(B)** Survival time of patients. **(C)** Representative Western blot showing the expression of COX4-1 in GBM tumors. Citrate synthase (CS) was probed as a loading control. **(D)** Densitometric analysis of Western blot in C. **(E)** Digitonin-solubilized mitochondria from GBM tumors were subjected to BN-PAGE followed by complex I and IV IGA assays and complex III Western blot analysis.

Results from our recent prospective biomarker trial in GBM demonstrated that the combination of *MGMT* promoter methylation and low CcO activity within GBM tumors may be predictive of long-term survival in patients [55]. To address the clinical relevance of our above findings, we examined the expression of COX4-1 and SC assembly in a panel of six of the GBM specimens with *MGMT* promoter methylation: three specimens from patients with high CcO activity and short overall survival (OS; mean CcO activity, 10.88 ± 1.00; mean OS, 134 days) and three specimens from patients with low CcO activity and long OS (mean CcO activity, 1.66 ± 0.22; mean OS, 691 days; **Fig. 9A** and **B**). Interestingly, tumor expression of the COX4-1 isoform was strongly associated with the high CcO/short OS group. Although some expression of COX4-1 was detected in GBM specimens from the low CcO/long survival group, the mean expression level was significantly lower, with the expression barely detectable in two of the specimens (**Fig. 9C**).

To analyze whether the structural organization of the mitochondrial respiratory chain differs between the two GBM specimen groups, digitonin-solubilized mitochondria were subjected to BN-PAGE. In mitochondria from the tumors with high CcO activity, CcO was identified at varying copy numbers (n) in complex III-IV SCs (III_2_IV_n_) and in larger complex I-III-IV SCs (I_1_III_2_IV_n_). On the other hand, in mitochondria from the tumors with low CcO activity, the activity of complex I and CcO was below the limit of detection by IGA. Moreover, western blot analysis of complex III clearly showed the absence of SCs (**Fig. 9E**).

## DISCUSSION

Renewed interest in understanding the metabolic changes that occur during cancer progression and the development of therapeutic resistance has led to research findings that suggest metabolic pathways as druggable targets. However, information regarding the metabolic profile of GBM cells that become resistant to radiotherapy has remained sparse. Here, we report that the development of GBM radioresistance triggered by fractionated radiation associates with a switch from a predominately glycolytic phenotype to a predominately OXPHOS phenotype. Furthermore, this phenotype switch, which appears to be mediated by a change in the COX4 isoform expressed within CcO, affects mitochondrial respiratory chain function, and thereby reduces ROS production.

Since the description of the Warburg effect almost 100 years ago [56], the idea that cancer cells rely heavily on aerobic glycolysis because of decreased or damaged OXPHOS has predominated. However, studies during the last few years have demonstrated that OXPHOS has an essential role in tumor progression and resistance to therapy [16, 17]. Specifically, Zu and Guppy demonstrated that even in the presence of high glycolytic rates, OXPHOS remains the major source of ATP in cancer cells [57], and several reports have demonstrated that cancer stem cells rely mainly on OXPHOS to fulfill energy demands in different cancer types, including leukemia [58], pancreatic cancer [59], breast cancer [60], and GBM [6]. Similarly, it was recently reported that OXPHOS is crucial for preserving glioma stem cells [17, 61]. Moreover, a shift in cancer cell metabolism toward OXPHOS has been reported in chemo- and radioresistant cancer cells [10, 11, 18, 19].

Here, we used radiosensitive U251, D456, and Jx39 GBM cells and radioresistant U251, D456, and Jx39 cells derived by exposure to fractionated radiation to determine the changes in metabolic phenotype that accompany the development of radioresistance in GBM. Our assessment of the mitochondrial OCR and the cellular glucose consumption and lactate production rates in these GBM-pair lines revealed that the isogenic radioresistant cells were, in general, less glycolytic than were the parental radiosensitive cells and had a higher rate of mitochondrial ATP production. Furthermore, the mitochondrial reserve capacity, a measure of the ability of cells to resist oxidative stress [62], was higher in the radioresistant cells and correlated with lower O_2_^•-^ production levels.

Perhaps underlying these crucial differences in metabolic phenotype, we found that the mitochondrial respiratory chains are differentially organized in radiosensitive and radioresistant GBM cells. In particular, the proportion of CcO found as monomers was lower and the proportion associated with complex III in SCs (III_2_-IV_n_) and larger structures containing I_1_III_2_IV_n_ was higher in radioresistant cells. These differences correlate with changes in O_2_^•-^ production and respiration, with the more monomeric CcO associated with lower OCR and elevated O_2_^•-^ production in radiosensitive cells consistent with their more glycolytic metabolism. A further intriguing finding is that the large differences in mitochondrial O_2_^•-^ production and SC assembly between radiosensitive and radioresistant cells correlated with the up- and down-regulation of CcO regulatory subunit COX4-1. In fact, the COX4-2 isoform was almost exclusively expressed in the parental radiosensitive GBM cells, whereas the COX4-1 isoform was almost exclusively expressed in the radioresistant cells. Interestingly, COX4-1 knockdown in radioresistant cells impaired CcO activity and decreased the OCR, but increased O_2_^•-^ production. In contrast, COX4-1 overexpression in radiosensitive cells increased CcO activity and the OCR but decreased O_2_^•-^ formation. Since activation of mitochondrial respiration usually increases ROS production [62, 63], we posit that enhanced SC assembly promoted by COX4-1 overexpression represses ROS production by increasing the efficiency of electron transfer among complexes I, III, and IV (CcO).

Our results may help explain the different redox and bioenergetic features of radiosensitive and radioresistant cells: the expression of COX4-1 and the greater proportion of CcO assembled into SC may contribute to the higher respiration rate, OXPHOS phenotype and reduced O_2_^•-^ production of radioresistant cells. Our results agree with a previous report that demonstrated in murine brain cells that assembly of mitochondrial complexes into SCs can regulate mitochondrial ROS production that occurs mainly during the process of OXPHOS [31].

We previously demonstrated that high tumor expression of COX4-1 correlates in a significant manner with worse patient prognosis, whereas high COX4-2 expression does not, suggesting that COX4-1 may have a novel function that differs from that of COX4-2 [15]. Additionally, an exploratory analysis from our recent prospective biomarker trial in GBM showed that the combination of *MGMT* promoter methylation and low CcO activity within tumors may be a predictive marker for longer patient survival [55]. Here, we found that mitochondrial complexes were largely incorporated into SCs in GBM specimens with high CcO activity (high COX4-1 expression) obtained from patients with short OS. In contrast, complex incorporation into SCs was significantly reduced in GBM samples with low CcO activity (low COX4-1 expression) obtained from patients with long OS. This trend suggests that SC assembly in GBM cells is mediated by COX4-1 expression and contributes to reduced OS in patients, possibly by minimizing ROS production; however, these observations must be validated in a much larger number of samples.

Many CcO subunits have been implicated in the assembly of mitochondrial SCs. The role of COX7A2L (SCAF1) in promoting SC assembly has been demonstrated in breast cancer [21], pancreatic cancer [64], normal mouse tissue [36, 65–67] and bovine heart mitochondria [27]. Similarly, knockdown of human COX17, a copper chaperone involved in the formation of the binuclear copper center of CcO, affects assembly and SC organization of CcO in HeLa cells [68]. More recently, it was demonstrated that human lung adenocarcinoma cells co-opt the expression of the normally sperm-specific CcO subunit COX6B2 to promote CcO incorporation into SCs and increase OXPHOS efficiency [35]. Thus, our data indicating that COX4-1 expression enhances CcO activity and promotes SC assembly in radioresistant GBMs is not without precedent. A switch in CcO isoforms have been previously associated with resistance to oxidative stress and aggressive phenotype of cancers cells [11, 15, 35, 69–71].

Overall, our results support the view that the exposure of GBM cells to fractionated radiation promotes the expression of the CcO subunit isoform COX4-1, which in turn promotes mitochondrial OXPHOS and SC assembly while minimizing ROS production. As the use of inhibitors that specifically target OXPHOS has raised concerns regarding toxicity to non-cancer cells, our results point to new avenues for potential therapeutic interventions converging on the mitochondria.

## MATERIALS AND METHODS

### Cell lines

Glioma cells were cultured as we previously described [10, 15, 72]. Radioresistant cell lines (U251-RR, D456-RR, and Jx39-RR) were developed from their respective parental cell lines (U251, D456, and Jx39) by weekly exposure to a single radiation dose of 5 Gy for five-weeks. During this period cells received an accumulated dose of 25 Gy, which is equivalent to a biologically equivalent total dose in 2-Gy fractions (EQD2) of 34.6 Gy. Stable U251 and D456 cell lines ectopically expressing FLAG-tagged COX4-1 (COX4-1-U251 and COX4-1-D456, respectively) or the FLAG expression vector (vector-U251 and vector-D456, respectively) were generated as we previously described [15]. All electroporations were performed using a Gene Pulser Xcell Electroporation System (Bio Rad, Hercules, CA) as we previously described [15, 73]. Glioma cells were electroporated with a lentiviral vector carrying one of four unique 29mer shRNA constructs specific for human *COX4I1* or scramble control shRNA (OriGene Technologies, Rockville, MD; catalog # TL313764). To generate stably transfected cell lines, cells were selected with 5 μg/ml puromycin for at least three weeks. The generation of GBM cells overexpressing *COX4I1* was previously described [15]. Cells were authenticated by STR Profile and Inter-species Contamination testing (IDEXX BioAnalytics, Columbia, MO) and evaluated for mycoplasma contamination (The Iowa Institute of Human Genetics Genomics Division).

### Clonogenic survival assay

A total of 1 × 10^5^ cells were plated in 60 mm dishes and allowed to grow in culture media for 48 h. Cells were then irradiated with 2, 4, 6, or 8 Gy at room temperature. Ionizing radiation was delivered with a dose rate of 0.805 Gy/min using a 6000 Ci^137^Cs cesium irradiator (J.L. Shepherd, San Fernando, CA). Immediately after irradiation, cells were plated at low density (250-20,000 cells per dish), and clones were allowed to grow for 14 days. Clones were then fixed with 70% ethanol and stained with Coomassie blue for analysis of clonogenic survival. Individual assay colony counts were normalized to that of control, with at least three cloning dishes per condition, repeated in at least two separate experiments.

### Comet assay

Single-cell gel electrophoresis (comet assay) was performed using a comet assay kit (Trevigen, Gaithersburg, MD; catalog # 4250-050-K) according to the manufacturer's instructions. Slides were imaged using a BX-61 light microscope (Olympus, Center Valley, PA) and comet images were analyzed using CometScore software (TriTek Corp., Sumerduck, VA). The percentage of DNA in the comet tail was calculated as a measure of DNA damage.

### Mitochondrial preparation and functional studies

Mitochondrial fractions were prepared from cultured cells as we previously described [72]. Mitochondrial respiration assays using freshly isolated mitochondria were performed by measuring O_2_ consumption in a 2-channel respirometer (Oxygraph-2k with DatLab software; Oroboros Instruments, Innsbruck, Austria) as we previously described [10, 11, 15, 72]. Glucose uptake experiments were carried out as previously described [10, 11] using 2-(N-(7-nitrobenz-2-oxa-1,3-diazol-4-yl)amino)-2-deoxyglucose (2-NBDG; Life Technologies, Grand Island, NY; catalog # N13195). Lactate accumulation in the culture medium was determined using a fluorescence-based L-lactate assay kit from Cayman (Ann Arbor, MI) according to the manufacturer's instructions. Mitochondrial complex activities were determined, with results normalized to citrate synthase activity, as previously described [10, 55, 73].

### Mitochondrial membrane potential

Mitochondrial membrane potential was assessed using a JC-10 Mitochondrial Membrane Potential Assay kit (Sigma; catalog # MAK159) according to the manufacturer's instructions. Δψ_m_ values were expressed in arbitrary units. FCCP (10 µM, 15 min), was added to cells to define the Δψ_m_ depolarized values.

### Rate of ATP production

The rate of ATP production was measured as previously described [74]. Briefly, glucose-dependent, hexokinase-catalyzed ATP hydrolysis was coupled to glucose-6-phosphate dehydrogenase-catalyzed reduction of NADP^+^ to NADPH in a 1:1 stoichiometry. To measure ATP production, autofluorescence of NADPH (340/460 excitation/emission) was measured continuously at 30°C using monochromatic fluorescence (FluoroMax-3; Horiba Jobin Yvon, Edison, NJ). Rates of ATP synthesis were quantified by applying a standard curve generated from ATP titrations.

### Measurement of ROS

The intracellular level of O_2_^•-^ was determined using MitoSOX Red (Invitrogen, Waltham, MA) as we previously described [10, 11, 15]. Briefly, 2 × 10^5^ cells were incubated with 2 µM MitoSOX Red for 25 min at 37°C. Some cells were preincubated with FCCP (10 µM) or AA (10 µM) for 15 min as indicated. Fluorescence was analyzed by flow cytometry (510 nm excitation and 580 nm emission) and 10,000 events were obtained per sample. The data were then analyzed using FlowJov10 software. The generation of H_2_O_2_ was measured using 10 μM Amplex^TM^ Red (ThermoFisher Scientific, Waltham, MA, USA, Cat. # A12222) as we previously described [73].

### Western blot analysis

SDS-PAGE and immunoblotting were performed as we previously described [10, 11, 15]. Antibodies used for immunoblotting were as follows: anti-COX4-1, anti-COXI, anti-UQCRC2 (1:1000, ab14744, ab14705and ab14745, Abcam), anti-COX4-2 and anti-citrate synthase (1:1000, 11463-AP and 16131-AP, Proteintech Group).

### BN-PAGE

BN-PAGE was performed as previously described [75, 76]. Mitochondria (150 μg) were solubilized in 50 μl of NativePAGE Sample Buffer (4X; Invitrogen, catalog # BN20032) containing digitonin (Invitrogen, catalog # BN2006) at a digitonin/protein ratio of 4 g/g, on ice for 20 min. Solubilized proteins were supplemented with Coomassie brilliant blue G-250 (Invitrogen, catalog # BN2004) at a ratio of digitonin/Coomassie dye of 4:1. Samples were separated on 3–12% NativePAGE Bis-Tris gels (Invitrogen, catalog # BN2012BX10) and run with dark blue cathode buffer at 30 V for 1 h. The cathode buffer was then replaced with light blue buffer and the gels were run for 20 h at 30 V at 4°C. Dark blue cathode buffer was prepared by adding 50 mL of 20X NativePAGE Running Buffer (Invitrogen, catalog # BN2001) and 50 mL of 20X NativePAGE Cathode Buffer Additive (Invitrogen, catalog # BN2002) in 900 mL of deionized H_2_O. Light blue cathode buffer was prepared by adding 50 mL of 20X NativePAGE Running Buffer and 5 mL of 20X NativePAGE Cathode Buffer Additive in 945 mL of deionized H_2_O.

### Acquisition of tissue specimens

Brain tumor tissue was collected as we previously described [55]. All patients provided written informed consent to the surgical procedures and gave permission for the use of resected tissue specimens. The protocol for this study was approved by the Institutional Review Board for Human Use at the University of Iowa (IRB # 202202231).

## Abbreviations:

AA: – antimycin A,
CcO: – cytochrome c oxidase,
FCCP: – p-trifluoromethoxyphenylhydrazone,
GBM: – glioblastoma,
IGA: – in-gel activity,
MID: – mean inactivation dose,
OCR: – oxygen consumption rate,
OS: – overall survival,
OXPHOS: – oxidative phosphorylation,
SC: – supercomplex,
ROS: – reactive oxygen species.
